# JNJ-64619178 radiosensitizes and suppresses fractionated ionizing radiation-induced neuroendocrine differentiation (NED) in prostate cancer

**DOI:** 10.3389/fonc.2023.1126482

**Published:** 2023-03-07

**Authors:** Jogendra Singh Pawar, Md. Yusuf Al-Amin, Chang-Deng Hu

**Affiliations:** ^1^ Department of Medicinal Chemistry and Molecular Pharmacology, Purdue University, West Lafayette, IN, United States; ^2^ Purdue University Interdisciplinary Life Sciences Graduate Program, Purdue University, West Lafayette, IN, United States; ^3^ Purdue University Center for Cancer Research, Purdue University, West Lafayette, IN, United States

**Keywords:** PRMT5, JNJ-64619178, neuroendocrine differentiation targeting, prostate cancer, Radiosensitization effect, DNA damage

## Abstract

**Background:**

Radiation therapy (RT) is a standard treatment regimen for locally advanced prostate cancer; however, its failure results in tumor recurrence, metastasis, and cancer-related death. The recurrence of cancer after radiotherapy is one of the major challenges in prostate cancer treatment. Despite overall cure rate of 93.3% initially, prostate cancer relapse in 20-30% patients after radiation therapy. Cancer cells acquire radioresistance upon fractionated ionizing radiation (FIR) treatment, eventually undergo neuroendocrine differentiation (NED) and transform into neuroendocrine-like cells, a mechanism involved in acquiring resistance to radiation therapy. Radiosensitizers are agents that inhibit the repair of radiation-induced DNA damage. Protein arginine methyltransferase 5 (PRMT5) gets upregulated upon ionizing radiation treatment and epigenetically activates DNA damage repair genes in prostate cancer cells. In this study, we targeted PRMT5 with JNJ-64619178 and assessed its effect on DNA damage repair gene activation, radiosensitization, and FIR-induced NED in prostate cancer.

**Methods:**

γH2AX foci analysis was performed to evaluate the DNA damage repair after radiation therapy. RT-qPCR and western blot were carried out to analyze the expression of DNA damage repair genes. Clonogenic assay was conducted to find out the surviving fraction after radiation therapy. NED was targeted with JNJ-64619178 in androgen receptor (AR) positive and negative prostate cancer cells undergoing FIR treatment.

**Results:**

JNJ-64619178 inhibits DNA damage repair in prostate cancer cells independent of their AR status. JNJ-64619178 impairs the repair of ionizing radiation-induced damaged DNA by transcriptionally inhibiting the DNA damage repair gene expression and radiosensitizes prostate, glioblastoma and lung cancer cell line. It targets NED induced by FIR in prostate cancer cells.

**Conclusion:**

JNJ-64619178 can radiosensitize and suppress NED induced by FIR in prostate cancer cells and can be a potential radiosensitizer for prostate cancer treatment.

## Graphical Abstract

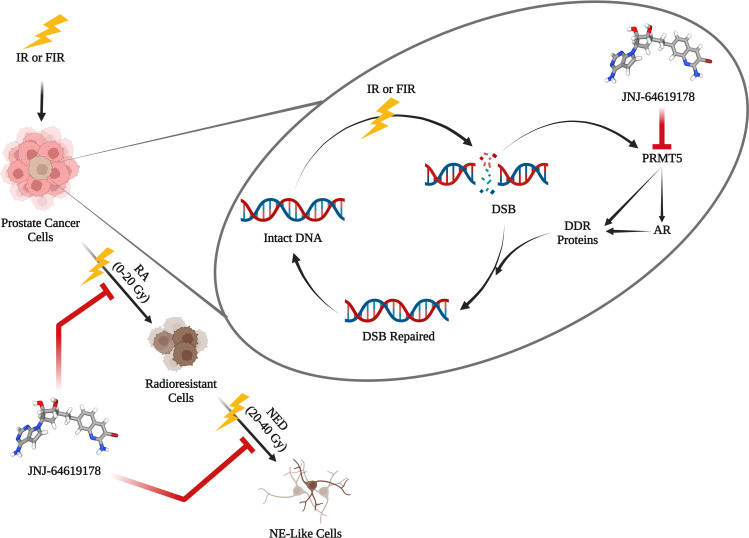

## Introduction

Prostate cancer is the second most frequent cancer among American men after lung cancer, with an expected 288,300 new cases and 34,700 deaths from the disease in 2023 (1). A common prostate cancer treatment plan includes radiation therapy, where high-energy radiation is used to eliminate cancer cells and reduce tumor size (2). It can be utilized either alone or in conjunction with other forms of treatment, such as chemotherapy and/or surgery (3). One of the most difficult aspects of treating prostate cancer is the recurrence of the disease after radiotherapy. This is partly because of the radioprotective mechanisms in cancer cells (3). Cancer cells employ different strategies to evade the effects of radiation therapy and thereby develop radioresistance, which critically contributes to tumor recurrence and metastasis (4, 5). Radioresistance is an acquired phenomenon in which cancer cells adapt to radiotherapy-induced changes through multiple mechanisms (6). Upon fractionated ionizing radiation (FIR) therapy, prostate cancer cells undergo neuroendocrine differentiation (NED) and thereby transdifferentiate into neuroendocrine-like (NE-like) cells and acquire radioresistance (7–11). In prostate cancer, some prostatic luminal cells switch their lineage from adenocarcinoma to neuroendocrine (NE)-like cells through transdifferentiation as a mechanism of developing resistance after treatment with ionizing radiation (12). NE-like cells are called so because they exhibit NE characteristics such as long neurite outgrowths and express NE markers including synaptophysin (SYN), chromogranin A (CgA), neuron-specific enolase (NSE) etc. These NE-like prostate cancer cells are usually resistant to existing treatments and correlates to poor prognosis (13). The principal mechanism of ionizing radiation (IR) to induce cellular apoptosis is through damaging DNA. Hence, one of the crucial adaptations in cancer cells is to acquire radioresistance *via* enhanced activation of DNA damage repair (DDR) pathways (5). This mechanistic insight paved the way to increase the vulnerability of cancer cells to radiation therapy by interfering with DDR using suitable radiosensitizers, drugs that enhance the extent of the lethal effect of radiation therapy (4).

Androgen receptors (AR) are vital for the growth, development, and proliferation of prostate cancer cells (14). AR promotes DNA damage repair in prostate cancer cells after radiation therapy and targeting AR pathway using AR signaling inhibitors (ASI) like Enzalutamide (Enz), Bicalutamide (Bic) are evolving as radiosensitization approaches in the management of prostate cancer (15–17). Prostate cancer cells can also repair DNA damage after radiation therapy independent of AR pathways (13). Since ASIs act by targeting AR signaling, thus cannot target AR-independent DNA repair mechanisms. So, there is an unmet need for drugs that can target the DNA repair pathways in IR-induced damaged DNA to radiosensitize prostate cancer cells in AR independent manner.

Protein arginine methyltransferase 5 (PRMT5) epigenetically activates the AR gene expression by symmetrically dimethylating arginine residue of histone H4R3 (H4R3me2s) at the AR gene proximal promoter region (18, 19). Additionally, following radiation therapy, PRMT5 gets upregulated and activates the expression of DNA damage repair (DDR) genes independent of AR (13). Upon IR-induced DNA damage, PRMT5 upregulates expression of genes involved in homologous recombination (HR) non-homologous end joining (NHEJ) DNA double-strand break (DSB) repair pathways. Targeting PRMT5 hinders the DSB repair by decreasing the HR and NHEJ genes expression after radiation therapy (13). The PRMT5 inhibitor JNJ64619178 (JNJ) binds to both the S-adenosylmethionine (SAM) and protein substrate-binding pockets. It is being tested in patients with advanced solid tumors and B cell non-Hodgkin lymphoma (NHL) that has relapsed or become resistant to treatment (20). In this study, we have investigated the potential of JNJ 64619178 to radiosensitize prostate cancer cells and to suppress FIR-induced NED in prostate cancer cells.

## Materials and methods

### Cell culture and treatment

LNCaP, DU145, and A549 cells were purchased from ATCC (Manassas, VA, USA). U87MG cells are received as a gift from Dr. Emily Dykhuizen. LNCaP cells were grown in RPMI-1640 medium, DU145 and U87-MG cells were grown in MEM medium, A549 cell were grown in Ham′s F12K medium (Corning, NY, USA), supplemented with 10% FBS (Atlanta Biologicals, Lawrenceville, GA, USA), 2 mM L-glutamine (Corning, NY, USA), and 100 units/mL penicillin and 100 μg/mL streptomycin (Gibco, Gaithersburg, MD, USA) at 37°C, with 5% CO_2_ in the humidified incubator. Cells were routinely passaged and were maintained for no more than 30 passages or 3 months.

### Ionizing radiation treatment

For IR treatment, X-RAD 320 biological irradiator was used (PXi Precision X-Ray, North Branford, CT, USA) with an average dose rate of ~2 Gy/60 sec. Control cells were ‘mock-irradiated’ by taking them out of the incubator for the same time as irradiated counterparts at room temp during treatment (21).

### Clonogenic assay

Clonogenic assay is considered as the gold standard for cancer cell radiosensitization studies, where surviving fractions are calculated from the number of colonies at the end of the experiment (22, 23). A lower surviving fraction indicates higher radiosensitization by the treatment/agent being tested. Clonogenic assay was performed to establish the surviving fraction post-IR treatment as described earlier (12). LNCaP, DU145, U87-MG, and A549 cells were seeded in 6 cm plates 24hr before the treatment to reach 70% confluency at the time of treatment. LNCaP cells were treated with 1 µM conc. of JNJ, Enzalutamide, Bicalutamide, or an equal volume of DMSO for 4 Days. DU145 cells were treated with 100 nM conc. of JNJ, Enzalutamide, Bicalutamide, or an equal volume of DMSO for 4 Days. A549 cells and U87-MG cells were treated with 10 µM conc. of JNJ or an equal volume of DMSO for 1hr each. After treatment, cells were exposed to indicated doses (0 Gy, 2 Gy, 4 Gy, 6 Gy, 8 Gy) of IR and immediately harvested, counted, and reseeded in 6 well plates in triplicates for Clonogenic assay. Cell culture media were changed after 7 days. After 14 days, the colonies were fixed and stained with crystal violet solution, and colonies were counted for calculating the surviving fraction. Those with >50 cells were considered as a colony. The cell numbers were optimized for each cell line for different radiation doses (LNCaP: 0 Gy-1,000 cells, 2 Gy-2,000 cells, 4 Gy-5,000 cells, 6 Gy-10,000 cells, and 8 Gy-20,000 cells) (DU145: 0 Gy-500 cells, 2 Gy-1,000 cells, 4 Gy-2,000 cells, 6 Gy-4,000 cells, and 8 Gy-8,000 cells), (A549: 0 Gy-500 cells, 2 Gy-1,000 cells, 4 Gy-2,000 cells, 6 Gy-5,000 cells, and 8 Gy-10,000 cells), (U87-MG: 0 Gy-100 cells, 2 Gy-200 cells, 4 Gy-500 cells, 6 Gy-1,000 cells, and 8 Gy-2,000 cells) (13).

### Targeting FIR induced NED

To assess the effect of targeting PRMT5 activity on FIR-induced NED of LNCaP and DU145 cells were seeded in 10 cm tissue culture dishes and were treated with JNJ-64619178 (1 µM for LNCaP cells and 100 nM for DU145 cells) or an equal volume of DMSO every 48hrs for 4 days before the IR treatment started. The treatment was replenished every 2 days thereafter and the control cells were treated with an equal volume of DMSO in place of JNJ-64619178 as shown in **Table 1**. Half of the media was changed once per week. Cells were treated with a cumulative dose of FIR (2 Gy each time, 5 days a week) until the total dose of 40 Gy FIR, and the cells were harvested 24hr after the final dose of IR. For assessing the effect of FIR on cells with and without JNJ-64619178 treatment, cells were imaged, harvested and cell viability was measured by trypan blue staining after 0 Gy, 10 Gy, 20 Gy, 30 Gy, and 40 Gy FIR treatment. Twenty-four hours following the last dose of IR, cells were imaged, harvested, used trypan blue staining to measure cell viability, and analyzed the cell images were to find out the cells (NED) with neurite length twice the cell body (LNCaP cells) and ≥3 neurites & neurite length twice the cell body (DU145 Cells). In the cell viability assay, lower cell viability in JNJ-64619178 treated groups compared to DMSO treated groups indicates higher radiosensitization of the cells. In neurites analysis, a lower number of cells with neurites in JNJ-64619178 treated groups in comparison to DMSO treated groups indicates inhibition of NED (21).

**Table 1:** FIR and JNJ64619178 (JNJ) treatment for targeting NED.

Group DU145/ LNCaP	Week 1 (0-10 Gy) FIR days 1-5	Week 2 (10-20 Gy) FIR days 1-5	Week 3 (20-30 Gy) FIR days 1-5	Week 4 (30-40 Gy) FIR days 1-5
1) Control	DMSO	DMSO	DMSO	DMSO
2) RA	Days 1,3,5 **JNJ**	Days 1,3,5 **JNJ**	DMSO	DMSO
3) NED	DMSO	Day 5 **JNJ**	Days 1,3,5 **JNJ**	Days 1,3,5 **JNJ**
4) Both	Days 1,3,5 **JNJ**	Days 1,3,5 **JNJ**	Days 1,3,5 **JNJ**	Days 1,3,5 **JNJ**

*JNJ represents JNJ-64619178.

### Trypan blue staining cell viability assay

To measure the change in cell viability after the treatment, cells were washed with PBS and detached by trypsinization. Cells were kept in a 37°C incubator for trypsinization for 5 mins and were resuspended in fresh medium. The cell pellets were collected after centrifuging them at 400 g for 5 mins at 4°C, the supernatants were discarded, and cold PBS was added to resuspend the cells. Cells were then aliquoted in small volumes and trypan blue was used to stain in a 1:1 ratio to count the number of viable cells in each sample using Countess™ II FL reusable slides and the countess™ II cell counter (Thermo Fisher, Waltham, MA, USA). In total, 4 readings were taken for each sample and the averages (cells/mL) were multiplied by the respective volume of each cell suspension to get the total number of living cells per treated sample. The total number of living cells after FIR treatment were normalized to IR- sample (21).

### Cell imaging

Before harvesting the cells, phase contrast images were taken for further NED analysis and neurites length measurements at 10x, and 20x objectives using Nikon TE2000 inverted microscope (Nikon Instruments Melville, NY, USA). Phase contrast images were taken and analyzed for changes in cellular morphology and NE-like cells (21).

### γH2AX staining and fluorescence imaging

For the γH2AX staining, cells were grown on a coverslip, were fixed with 3.7% formaldehyde. Permeabilized with 0.2% triton-X 100 for 5 mins, washed with cold PBS 3 times, and then blocking was done with 5% skimmed milk, 1hr at RT. Incubated with primary γH2AX antibody (Millipore) overnight at 4°C. Incubated with FITC conjugated anti-mouse secondary antibody (Jacksons) for 1hr in dark. After secondary incubation, the cells on coverslips were washed again with 0.1% PBST 3 times for 5 mins each and then mounted on glass slides with Prolong Antifade reagent (Invitrogen Molecular Probes, Eugene, OR, USA). Fluorescent images were captured with Nikon TE2000 inverted microscope at 60X objective (Nikon Instruments Melville, NY, USA). The number of foci/cell were counted and recorded for each cell individually. Foci in at least 60 cells were counted per biological replicate (BR), and the average number of foci/cell in each BR was further analyzed to determine the average number of foci/cell in 3 BR (13, 24–26).

### RT-qPCR

For quantifying the mRNA level for change in the expression of individual genes RT-qPCR was done. LNCaP cells were treated with 1 µM conc. of JNJ or an equal volume of DMSO for 4 Days. DU145 cells were treated with 100 nM conc. of JNJ or an equal volume of DMSO for 4 Days. A549 cells and U87-MG cells were treated with 1 µM conc. of JNJ or an equal volume of DMSO every 48hrs for 4 days. After treatment cells were exposed to 2 Gy of IR and left to recover the damaged DNA for another 6hrs before harvesting. Briefly, after the treatment, 1 ml of trizol reagent was added to each plate, incubate for 5 mins and then cells were collected in eppendorf for RNA extraction. RNA was purified and cDNA was prepared using the High-Capacity cDNA Reverse Transcription Kit (Promega) as per the instructions. The qRT-PCR analysis of HR and NHEJ genes was done as described earlier (27). List of qPCR primers is available as **Supplementary Material**. Fold changes greater than 1.0 indicates upregulation, while less than 1.0 indicates downregulation of mRNA transcription (18).

### Cell lysate preparation and western blotting

Cells lysate Preparation and Western Blotting was performed as per the protocol (28, 29). Briefly, LNCaP cells were treated with 1 µM conc. of JNJ, Enzalutamide, Bicalutamide, or an equal volume of DMSO for 4 Days. DU145 cells were treated with 100 nM conc. of JNJ, Enzalutamide, Bicalutamide, or an equal volume of DMSO for 4 Days. RIPA buffer was used to lyse the cells. Bradford assay was done to estimate the total protein conc. in each sample. Acrylamide gel (10 or 15%) was used to run SDS PAGE. To each well, 30/40 µg of protein was loaded and run at 120 V. After the run proteins on the gel were transferred to the nitrocellulose membrane at 100 V for 70 mins. Incubated in primary antibodies and followed by secondary anti-mouse or anti-rabbit secondary antibodies. After secondary antibody incubation, the blots were washed again with 0.1% PBST 3 times for 5 mins each. List of antibodies used is available as **Supplementary Material**. The blots were scanned with Odyssey^®^ DLx Imaging System and quantified and analyzed with image studiolite2 (28, 29).

### Statistical analysis

For statistical analyses, GraphPad Prism 9.00 for Mac (GraphPad Software, La Jolla California USA, www.graphpad.com) was used.

## Results

### JNJ-64619178 impairs repair of IR-induced DNA damage in prostate cancer cells independent of their AR status

**Figure 1:**
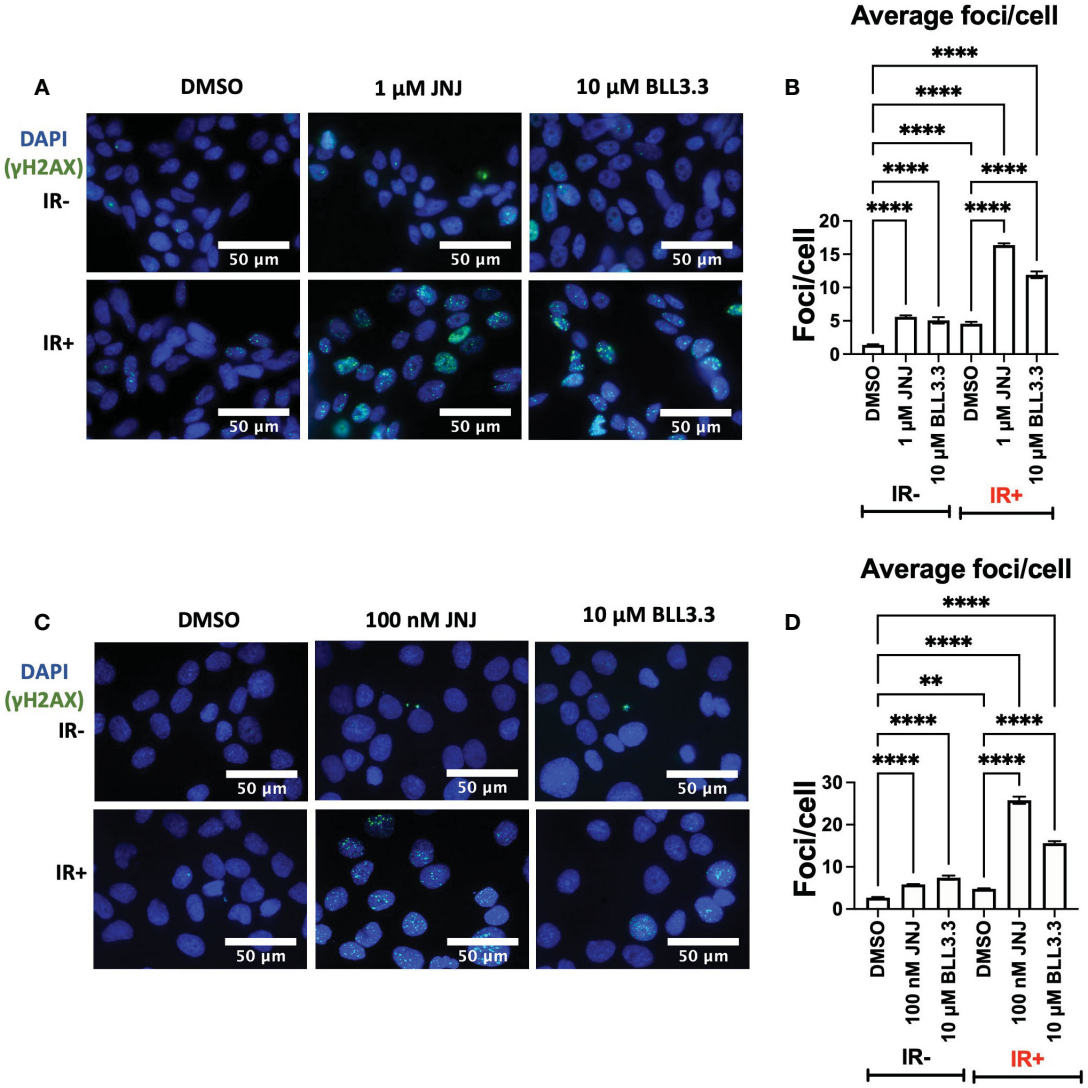
JNJ-64619178 hinders the radiation-induced damaged DNA repair in prostate cancer cells. **(A)** Representative fluorescence immunocytochemistry images showing double-stranded DNA breaks (γH2AX foci) images post 6hrs of 2Gy IR in LNCaP Cells. **(B)** Quantification of average DNA breaks per cell from **(A, C)** Representative fluorescence immunocytochemistry images showing double-stranded DNA breaks (γH2AX foci) images post 6hrs of 2Gy IR in DU145 Cells. **(D)** Quantification of average DNA breaks per cell from **(B)** For statistical analysis one-way ANOVA was performed with the Tukey test for multiple analysis, each bar in B, D are mean of SD of n=3 independent experiments (*P ≤ 0.05; **P ≤ 0.01; ***P ≤ 0.001; ****P ≤ 0.0001; NS, P > 0.05).

JNJ-64619178 is a small molecule inhibitor of PRMT5 (20). PRMT5 gets upregulated and promotes DDR upon radiation treatment in prostate cancer cells (13). Analyzing and quantifying the γH2AX foci is an established way of assessing the presence of DSBs and thereby DDR in cells, where a larger number of foci/cell indicates more DSBs and less DDR (26). To assess γH2AX foci, LNCaP (AR+) and DU145 (AR-) prostate cancer cells were treated with JNJ-64619178 for 4 days, then the cells were exposed to 2 Gy of X-ray (IR+) for inducing DSBs, next the cells were incubated for 6hrs to let them employ repair proteins at the site of DSBs (22). In both LNCaP & DU145 cells, higher γH2AX foci were observed in cells treated with JNJ-64619178+IR than DMSO+IR as shown in the fluorescent microscopic images (**Figures 1A, C**). The quantified data for DMSO+IR treated cells show a relatively lower number of γH2AX foci/cell indicating that the majority of DSBs were successfully repaired within the given 6hr time period (**Figures 1B, D**). Whereas, in the cells treated with JNJ-64619178+IR, a remarkably higher number of γH2AX foci/cell indicates the existence of a significantly higher number of DSBs, which was higher compared to the BLL3.3+IR (positive control) treated cells (**Figures 1B, D**). This suggests that JNJ-64619178 significantly reduced DDR in comparison to both DMSO and BLL3.3 in LNCaP & DU145 cells. Interestingly, the IR- cells treated with JNJ-64619178 alone were observed to have more γH2AX foci/cell than the DMSO-treated cells implying that JNJ-64619178 can hinder even basal level of DDR.

### JNJ-64619178 impairs DDR in prostate cancer cells by downregulating the expression of DSB repair proteins *via* inhibition of H4R3me2s histone methylation

**Figure 2:**
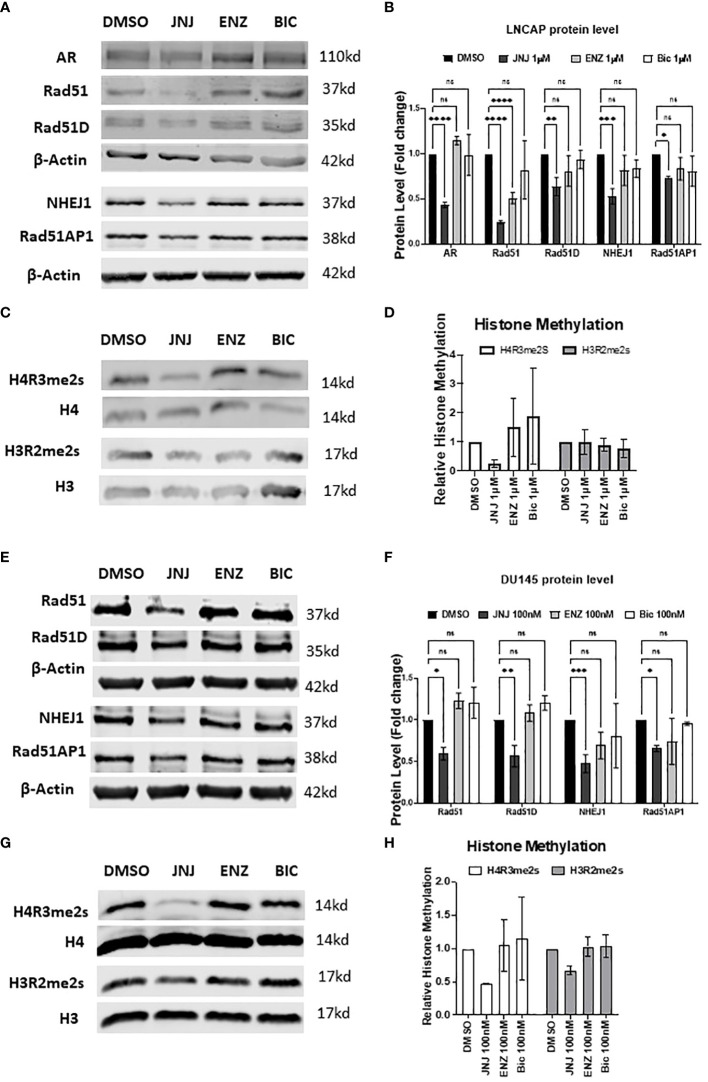
JNJ-64619178 targets DNA damage repair proteins expression and epigenetic methylation of histone proteins. **(A)** Representative western blot images showing the change in DNA damage repair proteins expression level compared with standard ASI, Enzalutamide (Enz), and Bicalutamide (Bic) after 4 days of treatment in LNCaP cells. **(B)** Histogram showing quantification of western blots shown in **(A)** For each biological replication, proteins were normalized to the beta-Actin to calculate the change in protein level after treatment **(C)** Representative western blot images of histone proteins and their methylation status post-treatment in LNCaP cells. **(D)** Histogram showing the change in the histone protein methylation post-treatment normalized to the specific histone level, from **(C, D)** Representative western blot images showing the change in DNA damage repair proteins expression level compared with standard ASI, Enzalutamide (Enz) and Bicalutamide (Bic) after 4 days of treatment in DU145 cells. **(E)** Histogram showing quantification of western blots shown in **(D)** For each biological replication, proteins were normalized to the beta-Actin to calculate the change in protein level after treatment **(F)** Representative western blot images of histone proteins and their methylation status post-treatment in DU145 cells. **(G)** Histogram showing change in the histone protein methylation post-treatment normalized to the specific histone level, from **(F)** For statistical analysis one-way ANOVA was performed with the Tukey test for multiple analysis, each bar in **(B, D, F, H)** are mean of SD of n=3 independent experiments (*P ≤ 0.05; **P ≤ 0.01; ***P ≤ 0.001; ****P ≤ 0.0001; NS, P > 0.05).

It’s now evident that JNJ-64619178 can impair DSB repair in prostate cancer cells. It has also been reported that PRMT5 is an epigenetic activator of the Androgen receptor (AR) which in turn promotes the DDR proteins expression (13, 19). Therefore, we compared the effect of JNJ-64619178 on the expression of DDR proteins in prostate cancer cells with standard Androgen Signaling inhibitors (ASIs) like Enzalutamide (ENZ), and Bicalutamide (BIC). We performed western blot analysis after treating LNCaP and DU145 prostate cancer cells with JNJ-64619178, ENZ, BIC, or DMSO for 4 days. JNJ-64619178 was found to be the most potent in terms of decreasing the expression of HR (RAD51, RAD51D, RAD51AP1) & NHEJ (NHEJ1) proteins compared to the DMSO control. Hence, JNJ-64619178 impairs DSB repair by downregulating the expression of HR and NHEJ repair proteins in both LNCaP (**Figures 2A, B**) and DU145 cells (**Figures 2E, F**).

Next, we investigated JNJ-64619178 mediated suppression of DSB repair proteins expression with the level of methylated histones since PRMT5 regulates AR and DSB repair proteins expression through symmetric dimethylation (SDMA) of H4R3 histone protein in the respective gene promoters (18, 19). To determine if JNJ-64619178 regulates these genes expression by targeting epigenetic methylation of the histone proteins, a western blot was performed for H4R3 (H4R3me2s) & H3R2 (H3R2me2s) histone methylation. Consistent with the changes in the DSB repair proteins after treatment, JNJ-64619178 significantly inhibited the H4R3me2s histone methylation in LNCaP (**Figures 2C, D**) and DU145 cells (**Figures 2G, H**) when normalized to the total H4 histone level, whereas no such changes were observed in the H3R2me2s histone methylation. These data collectively demonstrate that JNJ-64619178 inhibits the H4R3me2s histone methylation that downregulates the DDR protein level.

### JNJ-64619178 impairs the repair of radiation-induced DSBs in prostate cancer cells by suppressing DDR gene activation at the transcriptional level

**Figure 3:**
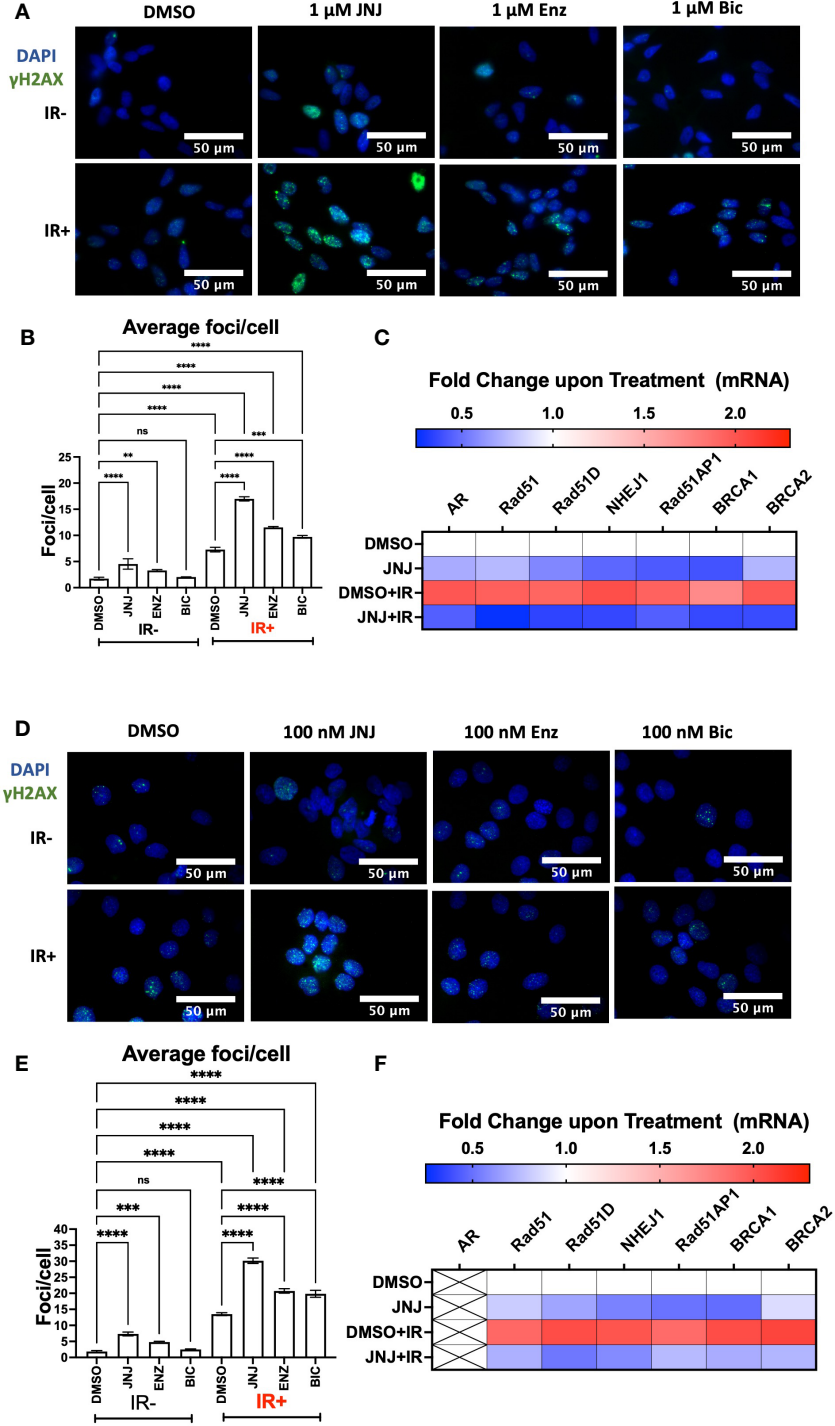
JNJ-64619178 impairs radiation-induced DSBs repair in both AR+ & AR- prostate cancer cells by targeting transcriptional activation of these genes involved in NHEJ and HR. **(A)** Representative fluorescence immunocytochemistry images showing double-stranded DNA breaks (γH2AX foci) images post 6hrs of 2Gy IR in LNCaP Cells. **(B)** Quantification of average DNA breaks per cell from **(A, C)** Histogram showing the change in NHEJ and HR genes mRNA at transcriptional level post 6hrs of 2Gy IR in LNCaP Cells. **(D)** Representative fluorescence immunocytochemistry images showing double-stranded DNA breaks (γH2AX foci) images post 6hrs of 2Gy IR in DU145 Cells. **(E)** Quantification of average DNA breaks per cell from **(D, F)** Histogram showing the change in NHEJ and HR genes mRNA at transcriptional level post 6hrs of 2Gy IR in DU145 Cells. For statistical analysis one-way ANOVA was performed with the Tukey test for multiple analysis, each bar in B, and E are mean of SD of n=3 independent experiments (*P ≤ 0.05; **P ≤ 0.01; ***P ≤ 0.001; ****P ≤ 0.0001; NS, P > 0.05).

AR promotes DNA damage repair in prostate cancer cells; hence researchers have proposed ASIs (ENZ; BIC) in impairing DDR to radiosensitize prostate cancer cells (17, 30, 31). To assess this, we compared the DDR-impairing potential of JNJ-64619178 with the AR signaling inhibitors (ASIs). Cells treated with JNJ-64619178+IR showed more γH2AX foci staining than cells treated with either ENZ+IR or BIC+IR. The quantified γH2AX foci/cell for JNJ-64619178+IR was found to be significantly higher than both ENZ+IR and BIC+IR in LNCaP (**Figures 3A, B**) and DU145 (**Figures 3D, E**) cells, which indicates that JNJ-64619178+IR impairs DDR better than ENZ+IR or BIC+IR. This implies that JNJ-64619178 impairs the repair of radiation-induced DSBs better than ASIs in prostate cancer cells independent of AR.

To gain further insight into JNJ-64619178 mediated regulation of HR and NHEJ proteins, we investigated if the regulation occurs at the transcriptional level. A decrease in HR and NHEJ mRNA levels was observed upon JNJ-64619178+IR treatment compared to DMSO control in both LNCaP and DU145 prostate cancer cells (**Figures 3C, F**). This confirms that JNJ-64619178 impairs DDR in IR-treated prostate cancer cells by suppressing DDR gene expression at the transcriptional level.

### JNJ-64619178 impairs DDR in lung cancer and glioblastoma cells by downregulating the transcription of DSB repair proteins

**Figure 4:**
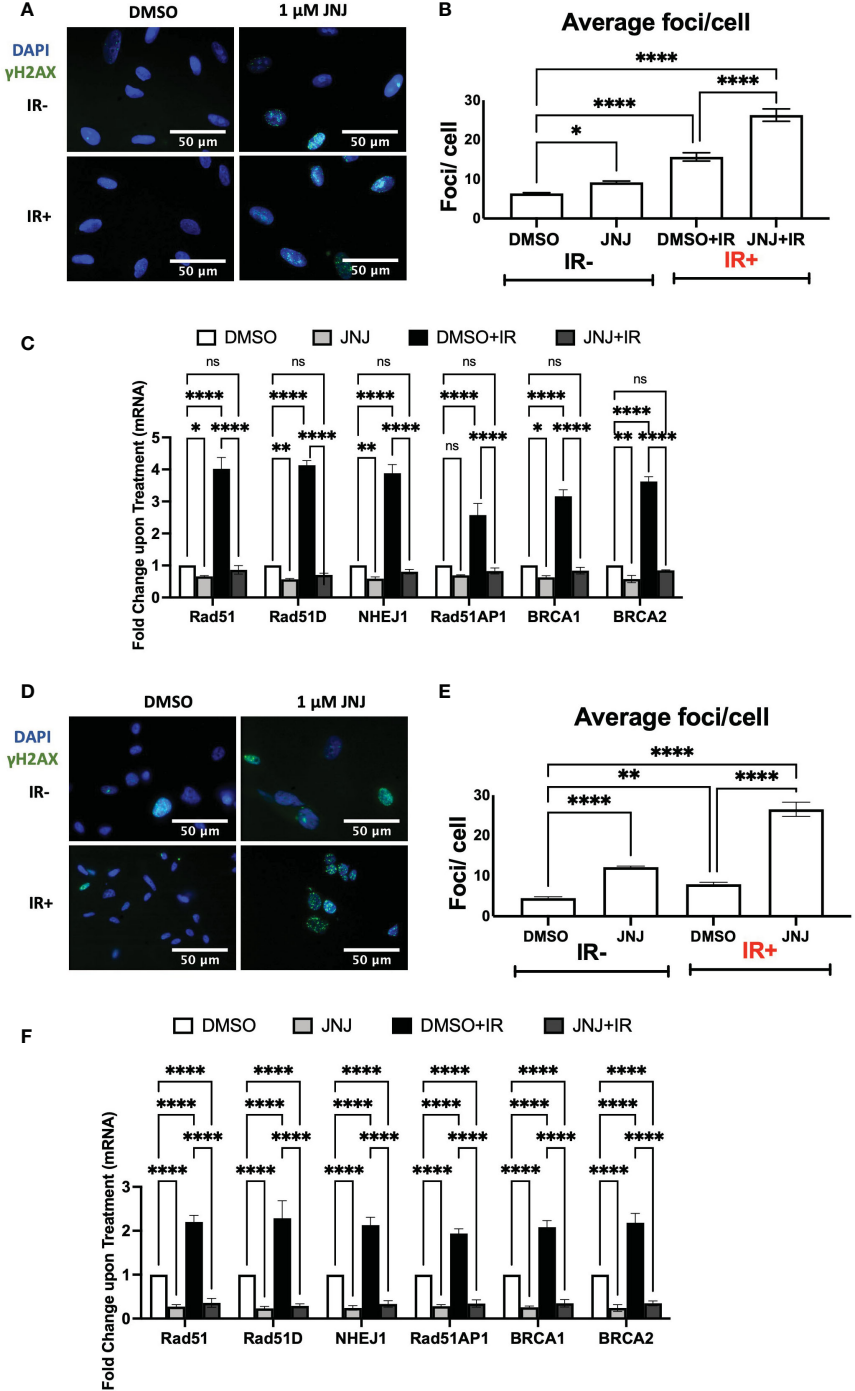
JNJ-64619178 inhibits double-strand break repair and DDR genes expression in A549 lung cancer and U87-MG glioblastoma cells. **(A)** Representative fluorescence immunocytochemistry images showing double-stranded DNA breaks (γH2AX foci) images post 6hrs of 2Gy IR in A549 Cells. **(B)** Quantification of average DSBs/cells from **(A, C)** Transcription level change in DDR gene expression as measured by the change in mRNA quantified by RT-qPCR in A549 cells post 6hrs of 2Gy IR treatment. **(D)** Representative fluorescence immunocytochemistry images showing double-stranded DNA breaks (γH2AX foci) images post 6hrs of 2Gy IR in U87-MG Cells. **(E)** Quantification of average DSBs/cells from **(A, F)** Transcription level change in DDR gene expression as measured by the change in mRNA quantified by RT-qPCR in U87-MG cells post 6hrs of 2Gy IR treatment. For statistical analysis one-way ANOVA was performed with the Tukey test for multiple analysis, each bar in **(B, C, E, F)** are mean of SD of n=3 independent experiments (*P ≤ 0.05; **P ≤ 0.01; ***P ≤ 0.001; ****P ≤ 0.0001; NS, P > 0.05).

To further extend the finding we assessed the DDR impairing potential of JNJ-64619178 in lung cancer (A549) and glioblastoma (U87MG) cells. The reasons for choosing these cells were (a) reports of frequent radioresistance in glioblastoma and lung cancer patients, and (b) PRMT5-mediated promotion of DNA damage repair in both A549 and U87MG cells (13, 32, 33). In both A549 & U87MG cells, JNJ-64619178+IR treated cells retained a significantly higher number of γH2AX foci compared to DMSO+IR treated cells (**Figures 4A, B, D, E**), which indicates that JNJ-64619178 impairs the repair of IR-induced DNA damages in these cells. Since it has been well established that cancer cells develop radioresistance by activating PRMT5-mediated upregulation of HR & NHEJ proteins upon IR treatment, plausibly we then investigated mRNA transcription of HR & NHEJ proteins in A549 & U87MG cells (13) In both A549 & U87MG cells, mRNA fold changes depicted that JNJ-64619178+IR treated cells significantly downregulated the transcription of HR (RAD51, RAD51D, RAD51AP1, BRCA1, BRCA2) & NHEJ (NHEJ1) proteins compared to DMSO+IR treated cells (**Figures 4C, F**). These findings suggest that JNJ-64619178 can impair the repair of IR-induced DNA damage in multiple cell types *via* transcriptional regulation of DSB repair gene expression.

### JNJ-64619178 sensitizes prostate and other cancer cells to ionizing radiation

**Figure 5:**
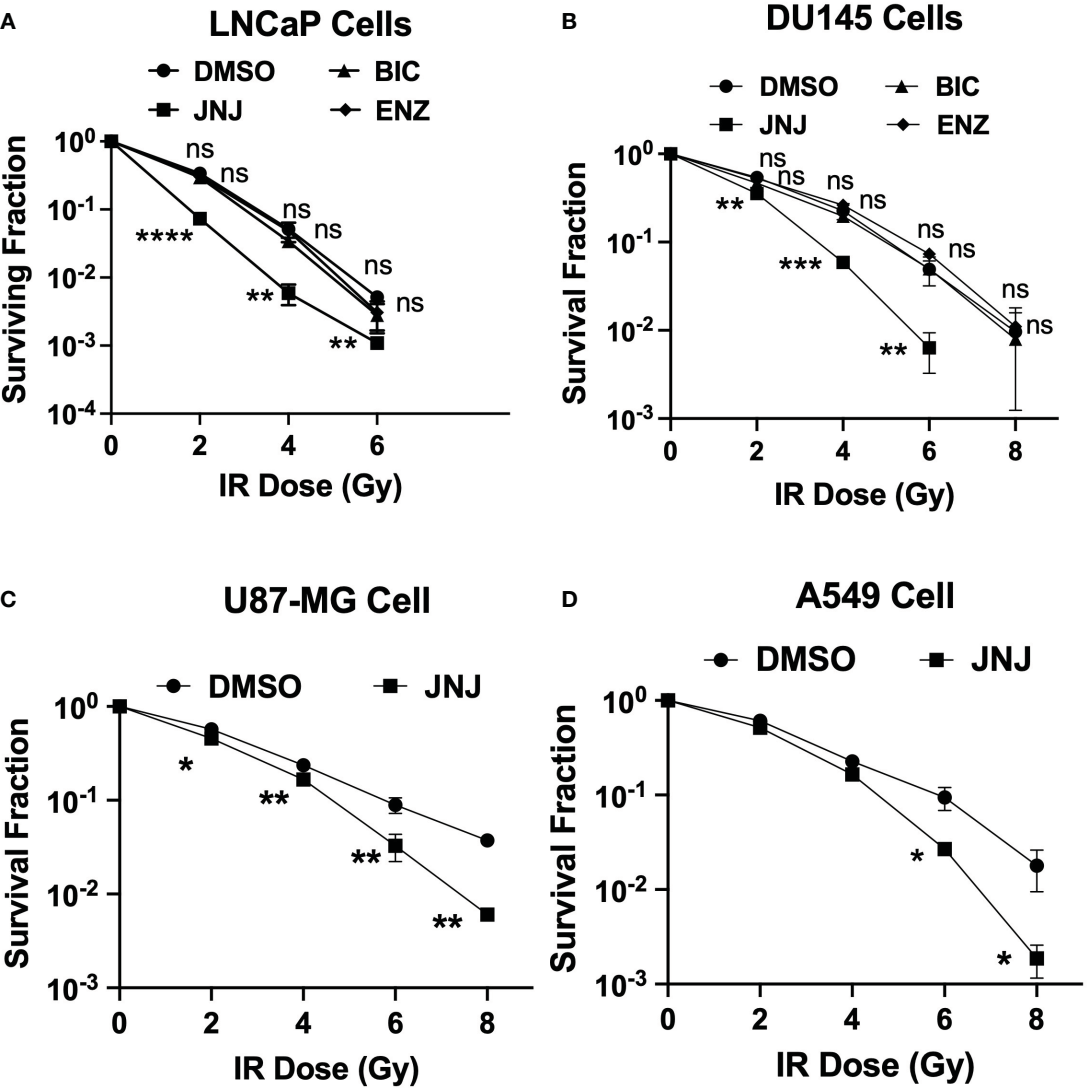
JNJ-64619178 radiosensitizes prostate and other cancer independent of AR. Surviving fraction quantification by Clonogenic assay in JNJ-64619178 treated cells immediately after indicated doses of IR treatment. **(A)** LNCaP Cells, **(B)** DU145 cells, **(C)** A549 cells, **(D)** U87-MG Cells. For statistical analysis one-way ANOVA was performed with the Tukey test for multiple analysis, each point in **(A-D)** are mean of SD of n=3 independent experiments (*P ≤ 0.05; **P ≤ 0.01; ***P ≤ 0.001; ****P ≤ 0.0001; NS, P > 0.05).

Next, we investigated the potential of JNJ-64619178 to sensitize prostate cancer cells to radiation therapy. The rationale behind this investigation was that (a) JNJ-64619178 impairs DDR, and (b) cancer cells develop radioresistance by upregulating DDR pathways (4, 13). We investigated the radiosensitization potential of JNJ-64619178 in LNCaP and DU145 prostate cancer cell lines. JNJ-64619178+IR treatment resulted in a significantly reduced surviving fraction compared to DMSO+IR treatment in both LNCaP (**Figure 5A**) and DU145 cells (**Figure 5B**). This suggests that JNJ-64619178 treatment can significantly sensitize prostate cancer cells to IR, while the radiosensitization potential of ASIs (ENZ, BIC) was found to be insignificant compared to DMSO control at these concentrations. Based on these observations, we extended the study to other frequently radioresistance-acquiring cancers like glioblastoma (U87MG cells) and lung (A549 cells) cancer (13, 33). Upon JNJ-64619178 treatment, both A549 and U87MG cell lines demonstrated a significantly reduced surviving fraction (**Figures 5B, C**). Taken together, these results indicate that JNJ-64619178 can sensitize prostate cancer and other cancer cells to ionizing radiation.

### JNJ-64619178 inhibits FIR induced NED

**Figure 6:**
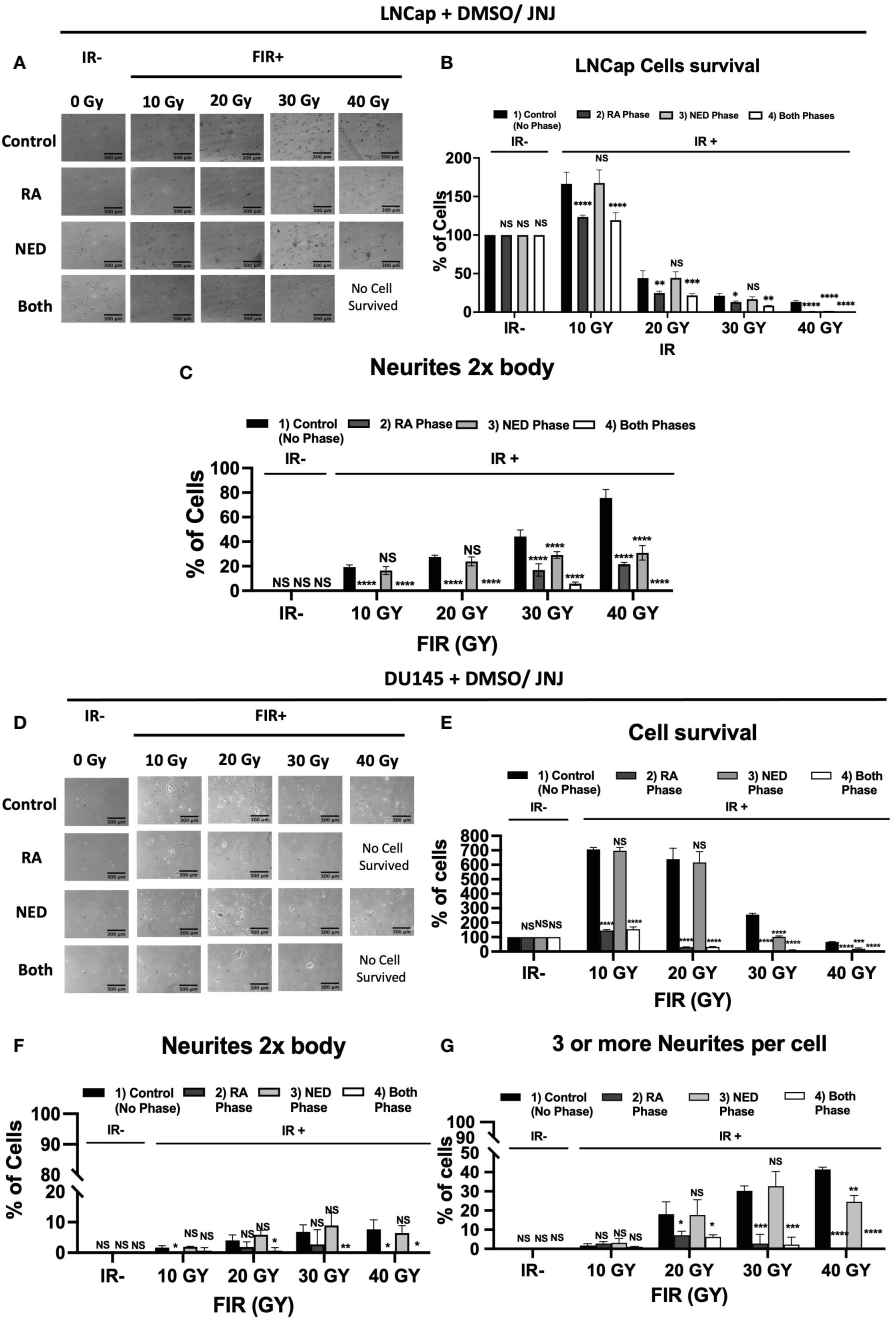
JNJ-64619178 inhibits FIR-induced Neuroendocrine differentiation (NED) in Prostate cancer. **(A–C)**, LNCaP cell treated with indicated cumulative doses of FIR (2Gy/dose, 5 doses/week). LNCaP cells were treated with JNJ-64619178 (1µM conc.) during the RA phase, NED phase, and both phases. **(A)** Phase-contrast images were captured 1 day after the last indicated dose of irradiation. **(B)** cell survival percentage. **(C)** NE-like cell (neurites 2x cell body length) percentage, calculated, analyzed, and presented. A similar experiment was done in the DU145 cell. **(D–G)** DU145 cells were treated with JNJ-64619178 (100 nM conc.) during the RA phase, NED phase, and both phases. **(D)** Phase-contrast images were captured 1 day after the last indicated dose of irradiation. **(E)** cell survival percentage. **(F)** NE-like cell (neurites 2x cell body length) **(G)** NE-like cell (more than 3 neurites/cell body) percentage, calculated, analyzed, and presented. For statistical analysis two-way ANOVA was performed, each bar in **(B–G)** are mean of SD of n=3 independent experiment (*P ≤ 0.05; **P ≤ 0.01; ***P ≤ 0.001; ****P ≤ 0.0001; NS, P > 0.05).

To acquire further insight into the impact of JNJ-64619178 on advanced radioresistant prostate cancer subtypes, we assessed the potential of JNJ-64619178 to inhibit different phases of FIR-induced NED. In both LNCaP & DU145 cells, JNJ-64619178 treatment during the RA phase (0-20 Gy) killed most of the cells and prevented the living cells from undergoing NED upon FIR treatment (**Figures 6A, B, D, E**). JNJ-64619178 treatment during the NED phase (20-40 Gy) reverted the NE-like morphology (neurites 2x cell body) and killed the cells that underwent NED in LNCaP (**Figures 6A, B**) & DU145 (**Figures 6D, E**) cells, respectively. Cells that were treated with JNJ-64619178 for all the phases of FIR (0-40 Gy), no cells survived compared to the control group in which 13.27% and 66.26% cells survived at the end of the experiment in LNCaP (**Figures 6A, B**) & DU145 (**Figures 6D, E**) cells, respectively. Morphological analyses further confirmed FIR-induced NED targeting by JNJ-64619178 treatment as evidenced by the changes in the percentage of cells with NE-like morphology after treatment during different phases of FIR treatment in both LNCaP (neurites 2x cell body) (**Figure 6C**) and DU145 cells (≥3 neurites & neurite length twice the cell body) (**Figures 6F, G**). Therefore, JNJ-64619178 can significantly inhibit FIR-induced NED and radiosensitizes prostate cancer cells.

## Discussion

RT is one of the first-line treatment strategies for localized prostate cancer (34). It causes DNA DSBs in cells, which are lethal if not repaired. Cancer cells can repair these DNA DSBs and regrow after RT as evidenced by the fact that 30-50% of high-risk and 10% of low-risk disease patients show cancer recurrence after RT (35–37). Radiation treatment induces a small set of prostate adenocarcinoma cell populations to develop radioresistance and undergo FIR-induced NED and transdifferentiate into NE-like cells (8, 21). Recently clinical significance of FIR-induced NED has been recognized as an important contributor to radioresistance and a significant factor for NE-like cell emergence (8, 12, 30, 38). Thus, there is a critical need for developing novel radiosensitizer drugs to improve the efficiency of radiation therapy and thereby ameliorate cancer mortality.

Radioresistance can be conferred by AR-induced activation of DDR genes in prostate cancer (31). Thus, combination therapies involving radiation with DDR targeting agents such as ASIs (Enz and Bic) are also used to radiosensitize prostate cancer (16, 39). As DDR activation can be mediated in both AR-dependent and independent manner, these ASIs cannot radiosensitize AR-independent cancer cells. It has been reported that DDR genes in prostate cancer cells get upregulated by PRMT5 upon radiation treatment independent of AR (13). PRMT5 is an oncogene, overexpressed in several human cancers, and is related to poor prognosis (40). Our lab has previously reported that PRMT5 cooperates with its cofactor PICln in the epigenetic upregulation of genes that take part in DDR and contribute to the radioresistance acquisition in prostate cancer cells. Hence, targeting PRMT5 can be a therapeutic strategy for the radiosensitization of prostate cancer. It is also shown to epigenetically upregulate the expression of the AR gene in prostate cancer cells. We also reported that prostate cancer cells require PRMT5 to repair IR-induced DNA damage independent of AR (13). Mechanistically, PRMT5 induces DDR genes (HR, NHEJ, G2 arrest) upon IR-induced DNA DSBs and therefore assists the cancer cells to recover and resist the effect of IR (19). Efforts are being made to improve the prostate cancer treatment efficacy by using radiosensitizers, to minimize the recurrence and improve prognosis.

JNJ-64619178 is a highly selective PRMT5 inhibitor (20). Thus, it was logical to anticipate that JNJ-64619178 will impair DDR in prostate cancer cells during radiation therapy. In this study, we demonstrated that therapeutic targeting of PRMT5 with JNJ-64619178 downregulates the expression of DSB repair proteins in prostate cancer cells. Interestingly, JNJ-64619178 impairs the repair of DSBs as shown by foci analysis (**Figures 1A, B**, **3A, D**, **4A, D**), even in the absence of external DNA break inducers. Here, we have shown that JNJ-64619178 can also downregulate the expression of genes that take part in DSB repair in HR and NHEJ pathways independent of AR, as shown in AR-independent DU145, A549, and U87-MG cell lines.

JNJ-64619178 can act as a radiosensitizer that targets PRMT5, which epigenetically inhibits the genes that take part in DSBs. Radiation therapy induces genotoxicity by inducing DNA damage, JNJ-64619178 inhibits the genes that take part in DSBs repair *via* both HR and NHEJ pathways. A higher percentage of radioresistance has been observed in patients with glioblastoma, prostate cancer, lung cancer, and a few other malignancies (33). Yang et al. described PRMT5-mediated DDR gene induction and radioresistance, and PRMT5 inhibition leading to radiosensitization in *in-vitro* and *in-vivo* lung cancer cells/models (41). Banasavadi-Siddegowda et al. depicted the vulnerability of glioblastoma cells to PRMT5 inhibition (42). So, it’s rational to expect that inhibiting PRMT5 may render cancer cells sensitive to ionizing radiation (IR) treatment. Hence, we decided to study radioresistance in the context of lung cancer (A549) and glioblastoma (U87-MG) cell lines. PRMT5 inhibition by JNJ-64619178 led to a significantly smaller number of colonies compared to vehicle (DMSO) treated groups in all the cancer cell lines (**Figure 5**), which indicates that JNJ-64619178 is successfully rendering the cancer cells sensitive to IR treatment.

We have also explored the ability of JNJ-64619178 in targeting FIR-induced NED and NE-like cells in prostate cancer since PRMT5 is required for NE-like cell survival. NED of prostate adenocarcinoma after radiation therapy is an evolving mechanism of radioresistance development in prostate cancer. Transdifferentiation of prostate cancer cells into NE-like cells occurs after radiation therapy. FIR induced NED is of high clinical relevance, is intrusive, and has a poor prognosis (7, 8, 10–12, 21, 30). We reported earlier that prostate cancer cells undergo NED and transdifferentiate into NE-like cells post FIR treatment with the cumulative treatment of 40 Gy (2 Gy/day, 5 days/week) and PRMT5 protein gets upregulated in response to FIR. FIR-induced NED appears to occur in two stages while cells undergo FIR treatment: First, acquisition of radioresistance (RA), and second, NED. During the RA phase (0-20 Gy) most of the cells died whereas the living cells start to show NE-like morphological characteristics and NE marker proteins (21). During the NED phase (20-40 Gy), NE-like morphological characteristics and protein increase. JNJ-64619178 treatment with FIR not only inhibited the FIR-induced NED but also killed all the cells after 40 Gy FIR treatment as compared to DMSO control cells. JNJ-64619178 inhibits the transdifferentiation of prostate adenocarcinoma *via* targeting FIR-induced NED to NE-like cells and has therapeutic potential as a radiosensitizing agent for prostate cancer radiotherapy. JNJ-64619178 will have added advantages of safety and bioavailability since the drug is already under Phase-I clinical trial for other indications (20). To the best of our knowledge, this is the first report demonstrating the potential of JNJ-64619178, PRMT5 inhibitor as a radiosensitizer, and FIR-induced NED inhibitor for prostate cancer treatment.

In summary, findings from this study strongly indicate that JNJ-64619178 radiosensitizes prostate cancer and targets the FIR-induced transdifferentiation of prostate adenocarcinoma and has therapeutic potential as a radiosensitizer for prostate cancer treatment. Future preclinical studies in suitable *in vivo* models will lead the way to clinical studies with JNJ-64619178 as a radiosensitizer in the management of prostate and other cancers.

## Data availability statement

The original contributions presented in the study are included in the article/**Supplementary Material**. Further inquiries can be directed to the corresponding author.

## Author contributions

JP: conceptualization, data curation, formal analysis, methodology, writing–original draft, writing–review and editing. MA-A: writing–review, and editing. C-DH: conceptualization, formal analysis, supervision, funding acquisition, methodology, writing–original draft, project administration. All authors contributed to the article and approved the submitted version.

## Supplementary material

Supplementary Figure 1 JNJ-64619178 hinders the radiation-induced damaged DNA repair in prostate cancer cells. **(A)** Representative fluorescence immunocytochemistry images showing double-stranded DNA breaks γH2AX foci, DAPI and Merged images post 6hrs of 2Gy IR in LNCaP Cells. **(B)** Representative fluorescence immunocytochemistry images showing double-stranded DNA breaks γH2AX foci, DAPI and Merged images post 6hrs of 2Gy IR in DU145 Cells.
