# Supplementary figures and images for: JNJ-64619178 radiosensitizes and suppresses fractionated ionizing radiation-induced neuroendocrine differentiation (NED) in prostate cancer
Source: Front Oncol. 2023 Mar 7;13:1126482. doi: 10.3389/fonc.2023.1126482 (PMC10028149; doi:10.3389/fonc.2023.1126482)

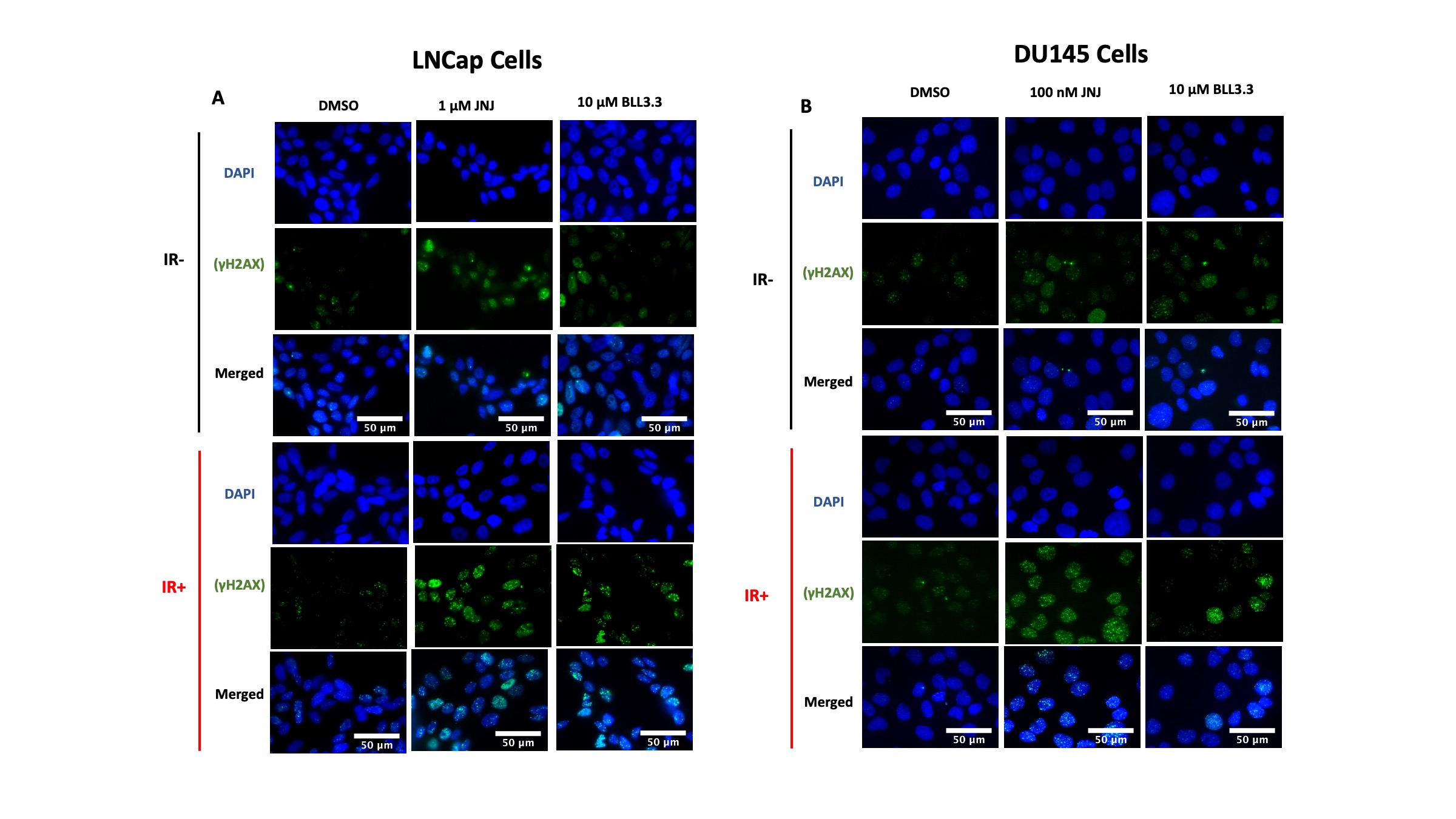

Supplement: Supplementary Figure 1 — JNJ-64619178 hinders the radiation-induced damaged DNA repair in prostate cancer cells. (A) Representative fluorescence immunocytochemistry images showing double-stranded DNA breaks γH2AX foci, DAPI and Merged images post 6hrs of 2Gy IR in LNCaP Cells. (B) Representative fluorescence immunocytochemistry images showing double-stranded DNA breaks γH2AX foci, DAPI and Merged images post 6hrs of 2Gy IR in DU145 Cells. [file Image_1.jpeg]
